# Manual on the proper use of meta-[^211^At] astato-benzylguanidine ([^211^At] MABG) injections in clinical trials for targeted alpha therapy (1st edition)

**DOI:** 10.1007/s12149-022-01765-1

**Published:** 2022-07-06

**Authors:** Naoyuki Ukon, Tatsuya Higashi, Makoto Hosono, Seigo Kinuya, Takahiro Yamada, Sachiko Yanagida, Masao Namba, Yoshihide Nakamura

**Affiliations:** 1 Advanced Clinical Research Center, Fukushima Global Medical Science Center, Fukushima Medical University, 1 Hikariga-oka, Fukushima, 960-1295 Japan; 2 Department of Molecular Imaging and Theranostics, Institute for Quantum Medical Science, National Institutes for Quantum Science and Technology, 4-9-1, Anagawa, Inage, Chiba, Chiba, 263-8555 Japan; 3 Department of Radiology, Faculty of Medicine, Kindai University, 377-2 Ohno-Higashi, Osaka-Sayama, Osaka 589-8511 Japan; 4 Japanese Society of Nuclear Medicine, 3-1-17 Nishi-Azabu, Minato-ku, Tokyo, 106-0031 Japan; 5 Atomic Energy Research Institute, Kindai University, Higashi-Osaka, 3-4-1 Kowakae, Osaka, 577-8502 Japan; 6 Japan Radioisotope Association, 2-28-45 Honkomagome, Bunkyo-ku, Tokyo, 113-0021 Japan; 7 Chiyoda Technol Corporation, 1-7-12 Yushima, Bunkyo-ku, Tokyo, 113-8681 Japan

**Keywords:** [^211^at] MABG, Targeted alpha therapy, Radiation protection, Pheochromocytoma

## Abstract

In this manuscript, we present the guideline for use of meta-[^211^At] astatobenzylguanidine ([^211^At] MABG), a newly introduced alpha emitting radiopharmaceutical to the up-coming World’s first clinical trial for targeted alpha therapy (TAT) at Fukushima Medical University in Japan, focusing on radiation safety issues in Japan. This guideline was prepared based on a study supported by the Ministry of Health, Labour, and Welfare, and approved by the Japanese Society of Nuclear Medicine on Oct. 5th, 2021. The study showed that patients receiving [^211^At] MABG do not need to be admitted to a radiotherapy room and that TAT using [^211^At] MABG is possible on an outpatient basis. The radiation exposure from the patient is within the safety standards of the ICRP and IAEA recommendations for the general public and caregivers or patient’s family members. In this guideline, the following contents are also included: precautions for patients and their families, safety management associated with the use of [^211^At] MABG, education and training, and disposal of medical radioactive contaminants. TAT using [^211^At] MABG in Japan should be carried out according to this guideline. Although this guideline is based on the medical environment and laws and regulations in Japan, the issues for radiation protection and evaluation methodology presented in this guideline are useful and internationally acceptable as well.

## Background of this safety management manual

In the field of nuclear medicine, radiopharmaceutical therapy, RI internal radiotherapy, or also known as targeted radioisotope/radionuclide therapy (TRT), is being actively carried out in recent years [1]. TRT is an internal radiotherapy using cytotoxic radionuclide-labeled pharmaceuticals. Based on the concept of “theranostics”, the medical indication of TRT is supposed to be decided by imaging diagnosis with PET / SPECT nuclei and after imaging, TRT is to be carried out by replacement of diagnostic nuclei to therapeutic nuclei. In addition, we have been embarking on a new era of TRT with alpha emitters in the last decade. Conventionally, only β-ray nuclides (^131^I, ^89^Sr, ^90^Y) have been used for TRT, but recently α-ray TRT have been clinically applied and are rapidly spreading. The α-ray nuclei TRT has received a great deal of attention due to its high therapeutic effect (Linear Energy Transfer / LET and high Relative Biological Effectiveness / RBE) [2]. After the approval by the European Medical Agency (EMA) in Europe and the Food and Drug Administration (FDA) in the United States in 2013, ^223^Ra chloride (trade name Xofigo) was approved for insurance in Japan in 2016 [3].

The ^223^Ra chloride was the world’s first α-ray TRT radiopharmaceutical clinically applied to treat bone metastasis of castration resistant prostate cancer and it showed an extension of prognosis that could not have been achieved with the conventional similar β-ray TRT agent, ^89^Sr chloride injection. Since insurance approval, it has become a blockbuster drug (a formulation boasting sales of 100 billion yen). In Japan, the number of ^223^Ra therapy treatments reached more than 4000 cases per year in 2017, the year after the approval in 2016, which is comparable to the treatment number of sodium iodide (^131^I) capsules for thyroid cancer [4]. It is expected that α-ray TRT will increase even further in the future.

In the treatment of malignant neuroendocrine tumors, effective chemotherapy has not been established to date. For these patients, TRT using beta-ray radiopharmaceutical, [^131^I] MIBG, has been performed. Since [^131^I] MIBG was not approved in Japan in the past, the private import of overseas manufactured drugs to only a few facilities in Japan was common. [^131^I] MIBG has been used as a standard therapy; however, [^131^I] MIBG with a conventional β-ray nuclei has not been effective in Japan [5]. In an observational study of 50 cases of malignant pheochromocytoma at a domestic facility by Yoshinaga et al., complete remission CR was 0 cases, partial remission PR was 1 case, stable SD was 40 cases, and advanced PD was 9 cases [5]. Recently, domestic manufacturing and marketing of [^131^I] MIBG was approved on September 27, 2021.

In Japan, the National Institute of Quantum Science and Technology (QST) has achieved results in several preclinical studies using animal models using ^211^At-labeled TRT agents. Meta- [^211^At] astatine-benzylguanidine ([^211^At] MABG) for malignant pheochromocytoma showed remarkable therapeutic effects [6]. Currently, Fukushima Medical University (FMU) and QST are preparing a first-in-human clinical trial of [^211^At] MABG as a collaboration study. Several other domestic institutions also have begun research and development of the ^211^At-labeled TRT radiopharmaceuticals.

As clinical trials of TRT using [^211^At] MABG are approaching, it is necessary to formulate release criteria for patients who received [^211^At] MABG from the radioisotope-controlled area, prior to the start of the clinical trial in FMU. The release criteria for the exit of patients who received radiotherapy drugs were stipulated by Pharmaceutical Safety No. 70 (June 30, 1998) for radioactive iodine (^131^I), and the cumulative dose calculation for caregivers and the public was calculated [7, 8]. The criteria are also necessary for α-ray nuclides, and they are needed to consider safe and appropriate doses for each nuclide and each radiopharmaceutical, and to calculate the cumulative dose for caregivers and the public. Osaka University has started a first-in-human clinical trial of [^211^At] NaAt for the treatment of metastatic thyroid cancer (ClinicalTrials.gov Identifier: NCT05275946), and Watabe, et al. already published a manual on the proper use of [^211^At] NaAt in clinical trials [9].

In this study, the dose of the new α-ray nuclide radiopharmaceutical, [^211^At] MABG, was evaluated with reference to the release criteria of the existing α-ray nuclide pharmaceutical, ^223^Ra chloride, and the related literature and reports were also reviewed. In preparing this clinical trial proper-use manual (1st edition) for nuclear medicine treatment using [^211^At] MABG injection solution, the following points were mainly examined: (1) establishment of safety management system in hospitals that use this drug, (2) appointment and role of radiation safety manager, (3) characteristics and pharmacokinetics of ^211^At and this drug, (4) the release of patients who received this drug, (5) evaluation of external and internal exposure doses from patients receiving this drug to caregivers and the public, (6) precautions for patients and their families after administration of this drug, (7) radiation safety management for patients using diapers and urinary catheters, (8) records and radiation measurement regarding the exit of patients who received this drug, (9) education and training for medical professionals involved in this clinical trial, (10) radiation protection and radioactive contamination prevention measures for medical staff, (11) disposal of medical radioactive contaminants (contaminated by ^211^At).

## Purpose of this safety management manual

Hospitalization in a dedicated radiotherapy room imposes a heavy mental and physical burden on the patient, and it is difficult for many medical institutions to maintain such a radiotherapy room due to cost reasons. Although [131I] MIBG treatment requires isolated hospitalization in a dedicated radiotherapy room, it has been clarified that astatine ([^211^At]), which is an α-ray nuclide with a short range, can be administered as an outpatient treatment [9].

This manual incorporates the points established in the Medical Care Act, and recommendations for radiological protection established by international organizations [10–14]; ideally, healthcare facilities that implement this treatment must follow the requirements covered in this manual to ensure radiation safety. The following points are summarized in this manual (safety management edition):

1. Guidelines for facility management.
2. Radiation exposure protection.
3. Storage and disposal of medical radioactive contaminants.

In addition, hospitals that implement this treatment must have achieved the following conditions regarding the standards of the implementing facilities to ensure the radiation safety:

(1) Hospitals that implement this treatment must meet the standards of protection from medical radiation stipulated in relevant laws and complete legal procedures.

(2) For this treatment, doctors and radiological technologists who have sufficient knowledge and experience regarding the handling of radiopharmaceuticals should work full-time, and doctors who have specialized knowledge and experience regarding the treatment of neuroendocrine tumors are also required to work full-time.

The purpose of this paper is to evaluate the pre-clinical and clinical measurement data from the QST and FMU, to examine the release criteria, and to prepare a user’s manual for the Phase 1 investigator-initiated clinical trial of [^211^At] MABG.

## Organizational initiatives in healthcare facilities that use [^211^At] MABG

Considering the unique characteristics of this drug, medical care teams in hospitals that carry out this treatment must perform this treatment. The medical care team is to consist of doctors, radiological technologists specializing in radiation safety management, and nurses specializing in patient care. This medical team should meet the requirements listed in the following sections.

## Structure and safety management in healthcare facilities

The hospitals that carry out this treatment have structural equipment for each of the rooms, specified in Article 30-8, Article 30-9, and Article 30-11 of the Medical Care Act Implementing Regulations. The medical facility must be approved by the prefectural governor, who has jurisdiction over the hospitals. For managers of healthcare facilities that perform this treatment, to ensure the safety of medical care, safe handling of [^211^At] MABG, and radiological safety, as stipulated in Article 1, item 3–2 of the Medical Care Act Implementing Regulations, there is a need to establish a safety management system for all healthcare personnel involved in administering this treatment.

## Assignment and roles of a radiological safety management supervisor and managers

The administrators of the hospitals that implement this treatment should ensure medical safety, safe handling of this drug, and safe radiation based on Article 1-11, clause 2, item 3-2 of the Medical Care Act Implementing Regulations. Accordingly, it is necessary to establish a systematic safety management system for doctors, radiological technologists, and others involved in this treatment.

The supervisors of the hospitals that implement this treatment must assign at least one or more radiological safety managers, as stipulated in Article 1-11, clause 2, item 3-2 of the Medical Care Act Implementing Regulations, and Japanese guidelines of radiation safety for therapeutic radiopharmaceuticals. These radiation safety managers shall supervise this treatment and its radiation safety and shall be involved in the implementation of related education and training of doctors involved in this treatment.

It is assumed that the following conditions are met when performing this treatment according to this manual.

1. Patients with malignant neuroendocrine tumors will be treated through administration of this drug.
2. Doctors with specialized knowledge of radiation safety management will explain the precautions for this treatment to the patient and/or family (caregivers) in advance. Based on this explanation, it will then be determined by the patient and/or family (caregivers) whether it is feasible for the patient to receive this treatment.
3. Flush toilets are to be installed in the residence before the patient returns home.
4. There are no restrictions on behavior in daily life, and individual patients can live independently without special assistance
5. Minimal contact between the patient and children or pregnant women for 3 days after administration of this drug must be ensured.

## Characteristics of ^211^At and [^211^At] MABG

The physical characteristics of ^211^At as a nuclide are shown in Table 1. ^211^At emits alpha rays with a physical half-life of 7.214 h. This radionuclide is produced by the ^209^Bi (α, 2n) ^211^At reaction. The astatine element is one of the halogens with atomic number 85. It is known that ^211^At decays into two types of progeny nuclides due to α decay and EC decay (Table 1), but ^211^Po decays to ^207^Pb, which is a stable nuclide with a half-life of 0.516 s, so the dynamics are considered to be almost the same as ^211^At. ^207^Bi is a long half-life nuclide with a half-life of 31.20 years.

**Table 1:** Physical properties of ^211^At, ^211^Po and ^207^Bi

Nuclide	Half-life	Decay mode	Maximum alpha energy (MeV) and emission rate	Main photon energy (MeV) and emission rate	Emission of internal conversion electrons per 100 disintegrations (%)	Effective dose rate constant (μSv m^2^ MBq^−1^ h − 1)
^211^At	7.214 h Progeny ^207^Bi _a_^211^Po	α EC	5.867–41.8% Other 58.20%	0.700–0.0035% 0.743–9.5 × 10^–4^% 0.687–0.26% 0.0787–31.1% Po-K_α_ 0.0906–8.5% Po-K_β_ 0.0124–18.9% Po-L	0.015	0.00580 0.00644^a^
^211^Po	0.516 s	α	6.569–0.54% 6.891–0.56% 7.450–98.9%	0.570–0.55% 0.898–0.56% Other 0.0744–0.016% Po-K_α_ 0.0875–0.0043% Po-K_β_ 0.0117–0.0084% Po-L	0.012 0.014	0.00110
^207^Bi	31.20 years Progeny^a^^207m^Pb	β+ EC	0.807–0.039% 100%	0.570–97.8% ^207m^Pb 1.064–74.6% ^207m^Pb 1.770–6.9% Other 0.0744–58.4% Po-K_α_ 0.0875–15.9% Po-K_β_ 0.0117–33.8% Po-L	2.1 9.7 0.031	0.202*

^a^Includes contribution from progeny nuclides, which is in radioactive equilibrium

Radiation exposure from the progeny nuclides (^207^Bi and ^207m^Pb) can be negligible because the half-life of ^207^Bi is 31.2 years and its biological half-life will be much earlier than that. Therefore, the radioactivity of the ^207^Bi (and progeny ^207m^Pb) produced in patient’s body is supposed to be extremely low

Source: Radioisotope Pocket Data Book (12th Edition) published by the Japan Radioisotope Association, 2020

The astatine element belongs to halogen like iodine in the periodic table and behaves similarly. It is speculated that astatine has several chemical forms such as At^−^, At^+^, At(OH)_2_^−^, AtO_2_^−^, AtO(OH)_2_^−^, and AtO^+^. The astatine element contained in this drug is distributed in the stomach, lungs, thyroid glands, salivary glands, testes, etc. via a sodium iodine symporter, etc., and is excreted in urine [15–18].

Using normal mice, the biodistribution after 5 min, 1, 3, 6, and 24 h of intravenous administration of [^211^At] MABG was evaluated by measuring the radiation dose and weight of each organ using an anatomical method. Furthermore, the residence time (h) was calculated from the area under the time radioactivity curve, and the residence time was entered into the internal exposure dose calculation software IDAC-Dose 2.1, assuming that the distribution in humans was the same as that in mice. Then, the absorbed dose (mGy/MBq) of each organ was estimated (Table 2) [18].

**Table 2:** Estimated absorbed dose after.^211^At-MABG in adult males [18]

Organ/tissue	Absorbed dose (mGy/MBq)
Brain	0.0068
Thyroid gland	1.140
Salivary gland	0.438
Myocardium	0.443
Lung	0.00924
Liver	0.137
Stomach	0.211
Small intestine	0.195
Colon	0.135
Kidney	0.115
Adrenal gland	0.517
Pancreas	0.0924
Spleen	0.182
Tastes	0.0678
Red bone marrow	0.00968

## Criteria for release of patients administered radiopharmaceuticals

The criteria for release (Notice No. 70 of Pharmaceutical Safety) were issued as guidelines for ensuring the QOL of patients treated with radiopharmaceuticals and ensuring the safety of the public and caregivers against radiation [7]. This was issued as an interpretation of the proviso stipulated in Article 30-15, clause 1 of the Medical Care Act Implementing Regulations. The outline of the release criteria is as follows:

1. Scope of application: when a patient who has been administered a radiopharmaceutical drug is released or returns home from a medical radioisotope room or radiotherapy room in a hospital.
2. Release criteria: safety standards have been established as "dose limit", as 1 mSv per year for general public, and 5 mSv per case for caregivers, in consideration of the benefits to both the patient and the caregiver shown in Table 3.

**Table 3:** Radioactivity level for release of patients who received administered radiopharmaceuticals

Radionuclides	Administered dose or residual radioactivity in the body (MBq)
Strontium-89	200^a^
Iodine-131	500^b^
Yttrium-90	1184^a^

^a^Maximum administered dose

^b^The radioactivity dose of iodine-131 is derived from the external dose from the patient's body plus the internal dose from inhalation of iodine-131 discharged with the patient's exhalation

Source: “Proper use manual for pain relief of painful bone metastases using strontium chloride (^89^Sr)”, “Proper use manual for radioimmunotherapy with Yttrium (^90^Y) Labeled Anti-CD20 Antibody” and “Proper use manual for internal therapy using iodine (.^131^I) sodium capsules” published by the Japanese Society of Nuclear Medicine

Specifically, if any of the following (a)–(c) applies, the patient is allowed to leave or return home.

(a) Release criteria based on dose.

If the dose or residual radioactivity in the body does not exceed the radioactivity shown in Table 3, leaving/returning home is permitted.

(b) Release criteria based on measured dose rate.

If the dose rate measured at a point 1 m from the patient's body surface does not exceed the values in Table 4, the patient is allowed to leave or return home. These reference values of (a) and (b) were calculated based on the dose, the physical half-life, the exposure coefficient of 0.5 at a point 1 m from the patient's body surface, and the 1-cm dose equivalent constant.

**Table 4:** Dose rate for release of patients who received administered radiopharmaceuticals

Radionuclides	1-cm dose equivalent rate at 1 m (ambient dose equivalent rate) from the patient’s body surface (μSv/h)
Iodine-131	30^a^

^a^The equivalent dose rate of iodine-131 is derived from the external dose from the patient's body plus the internal dose from inhalation of iodine-131 discharged with the patient's exhalation

Source: “Proper use manual for internal therapy using iodine (.^131^I) sodium capsules” published by the Japanese Society of Nuclear Medicine

(c) Release criteria based on cumulative dose calculation for each patient.

Based on the cumulative dose calculated for each patient, leaving/returning home is permitted in the following cases (Table 5).

**Table 5:** Cases that meet the release criteria based on the cumulative dose evaluation for each patient

Radionuclides	Scope of application	Administrated dose (MBq)
Iodine-131	Residual thyroid ablation after total thyroidectomy in differentiated thyroid cancer without distant metastasis^a^	1110^b^
Radium-223	Treatment of castration-resistant prostate cancer with bone metastasis^c^	12.1^d^ (72.6^e^)

^a^Limited to implementation in accordance with the guidelines prepared by related academic societies(“Outpatient treatment with I-131 (1110 MBq) for the purpose of residual thyroid destruction”)

^b^The radioactivity dose of iodine-131 is derived from the external dose from the patient's body plus the internal dose from inhalation of iodine-131 discharged with the patient's exhalation

^c^Limited when it is performed by administering 55 kBq/kg per dose of radium chloride (Ra-223) injection at 4-week intervals (up to 6 times) according to the implementation guidelines prepared by related academic societies (“Proper use manual for internal therapy using radium chloride (Ra-223) injection”)

^d^Maximum dose per administration

^e^Maximum dose per treatment

Source: “Outpatient treatment with iodine-131 (1,110 MBq) for the purpose of residual thyroid destruction”, and “Proper use manual for internal therapy using radium chloride (Ra-223) injection” published by the Japanese Society of Nuclear Medicine

(3) Release record.

If a patient is allowed to leave, the following must be recorded and kept for 2 years after release.

(a) Administered dose, release date and time, dose rate measured at the time of release.

(b) Contents of notes and guidance for mothers with breast-feeding infants.

(c) If release is permitted based on (c) of (2) in the preceding paragraph, the calculation method of the cumulative dose for which the release is permitted should be recorded.

Additional precautions are described as follows:

1. When allowing the patient to leave or return home, explain daily safety procedures in written form and verbally to avoid unnecessary exposure to third parties as much as possible.
2. If the patient has a lactating infant, give sufficient explanation, caution, and guidance.
3. Regarding protection according to the physical characteristics of radionuclides, explanations to patients and caregivers, and other safety management, refer to the guidelines prepared by organizations such as radiation-related academic societies.

## Factors related to the evaluation of exit criteria

The time of contact with the patient, the distance to the patient, and the radiation dose are factors of external exposure dose. Therefore, the exposure coefficient, which is a factor to be considered when evaluating the exposure dose of a third party, is set according to the degree of involvement with the patient.

(1) Exposure coefficient for caregivers: 0.5

It has been reported that 0.5 is a reasonable exposure coefficient for caregivers of patients who require careful nursing care, based on the measured doses of patients who received radiopharmaceuticals [19]. In addition, in a research study in Japan that measured the exposure dose from treated patients, it is appropriate to use an exposure coefficient of 0.5 [20]. Based on the above, 0.5 was adopted as the exposure coefficient in the dose evaluation of caregivers after the patient left and returned home.

(2) Public exposure coefficient: 0.25.

There is a report stating that it is appropriate to adopt an exposure coefficient of 0.25 based on the actual measurement values of the patient’s family exposure dose in ordinary households [19]. Hence, 0.25 was adopted as the exposure coefficient for family members other than the caregiver and other members of the public after the patient left and returned home.

## Exposure dose of a third party from patient administered [^211^At] MABG

The exposure doses of third parties such as caregivers and the public include both external exposure due to radiation emitted from radioactive substances in the body of patients receiving this drug and internal exposure due to contamination of the patient's excrement. The following is a combined evaluation of the dose exposed to a third party.

## Evaluation of the external exposure dose

Calculation formula for the dose rate of external exposure to a third party from a patient who received this drug, is as follows:

$$I = A \times C\times Fa / L2$$

Here, *I*: effective dose rate at the calculated evaluation point [μSv/h], *A*: residual radioactivity in the body of the treated patient [MBq], *C*: effective dose rate constant of ^211^At [μSv m^2^ MBq^−1^ h^−1^] (The value in Table 1, 0.00644 [μSv m^2^ MBq^−1^ h^−1^] is used), *Fa*: effective dose transmission rate (if there are multiple barriers, the value of the product of the transmission of each shield is taken as the total transmittance), *L*: distance from the radiation source to the evaluation point [m].

The cumulative dose of external exposure when continuously exposed to a third party at 1 m from the body surface of the patient who received this drug is calculated as follows: the cumulative dose to be exposed to a third party after leaving or returning home from a patient who received this drug is evaluated by the effective dose rate at 1 m from the patient's body surface. It is assumed that this drug is not excreted from the body and so only the physical half-life is considered.

(1) Caregiver exposure

Cumulative dose of external exposure = 540 [MBq/time] × 0.00644 $[\mathrm{\mu Sv}\cdot {\mathrm{m}}^{2}\cdot {\mathrm{MBq}}^{-1}\cdot {\mathrm{h}}^{-1}] \times 1.443 \times 7.214 [\mathrm{h}] \times 0.5 \, \times 4 [\mathrm{times}/\mathrm{treatment}] = 72.4 [\mathrm{\mu Sv }/\mathrm{ Treatment}]$

The following is to be noted:

540 [MBq/dose]: maximum dose of this drug per patient, 0.00644 [μSv m^2^ MBq^−1^ h^−1^]: effective dose constant of ^211^At, 1.443: coefficient for calculating life expectancy from the half-life of nuclides, 7.214 [h]: physical half-life of ^211^At, 4 [times/treatment]: maximum number of doses per year for treated patients, 0.5: the exposure coefficient of the caregiver.

(2) Exposure for general public

Cumulative dose of external exposure = 540 [MBq/time] × 0.00644 [μSv m^2^ MBq^−1^ h^−1^] × 1.443 × 7.214 [h] × 0.25 × 4 [times/treatment] = 36.2 [µSv/treatment].

It is to be noted that:

0.25: public exposure coefficient.

In both cases, the dose constraint value per caregiver is 5 mSv (5000 μSv), which is far below the public annual dose limit of 1 mSv (1000 μSv).

## Evaluation of the internal exposure dose

The excrement from patients treated with [^211^At] MABG may flow out into rivers through sewage treatment plants, mainly in the form of urine, and be used as drinking water after sewage processing. Therefore, in the estimation of the internal exposure dose, it is assumed that all the radioactivity administered to the patient will flow out to the river.

The public exposure dose will be examined using the Yodogawa River water system model in the Osaka area in Japan, where the utilization rate of purified treated water is high, as in the case of the previous examination of exit criteria [19]. Regarding the exposure dose of caregivers, since astatine is homologous to iodine, the caregiver's exposure dose is estimated referring to "Evaluation of the dose received by the caregiver from the patient administered with iodine-131" [19]. Based on the results of these studies, the recommendations of the International Commission on Radiological Protection (ICRP) and the safety standards of the International Atomic Energy Agency (IAEA) are satisfied as the annual dose limit of 1 mSv for the public and the dose constraint value of 5 mSv per caregiver.

The annual number of new cases of pheochromocytoma in Japan is reported to be about 1000 cases/year (1998). Among them, the rate of being diagnosed as malignant is 11%, and it is assumed that [^211^At] MABG therapy will be performed in all cases [21]. A maximum dose of 540 [MBq/person] is expected. It is said that treatment intervals in [^131^I] MIBG treatment are preferably at least 3–4 months apart. Therefore, assuming the same treatment protocol, the dose of [^211^At] MABG will be administered up to 4 times a year, and the amount used in the Osaka area will be 540 × 4 × 12.1 = 26,136 [MBq/year] (26.136 GBq/year) in terms of population ratio [19, 22].

To evaluate on the safe side, considering that all ^211^At administered to the patient flowed into the Yodogawa River system, the radioactivity concentration in the Yodogawa River system was 26,136 [MBq/year] ÷ 4.1 [T l/year] = 6.375 × 10^–3^ [Bq/l]. Here, 4.1 T liter is used as the annual average discharge of the Yodogawa River system (annual average from 1991 to 1995).

Annual ^211^At intake for a person from the general public (assuming 2 L of drinking water per day) is 6.375 × 10^–3^ [Bq/l] × 2 [l/day] × 365 [day/year] = 4.654 [Bq/year], and the internal exposure dose for one year in the above case is 4.654 [Bq/year] × 1.1 × 10^–5^ [mSv/Bq] × 1,000 [µSv/mSv] = 0.05 µSv.

Here, 1.1 × 10^–5^ [mSv/Bq] is the effective dose coefficient by oral ingestion of ^211^At [23]. This figure, 0.05 [µSv] is far below the public annual dose limit of 1 mSv (1000 µSv).

Since astatine is an element homologous to iodine and has similar chemical properties, the caregiver’s internal exposure dose is estimated by referring to “Evaluation of the dose received by the caregiver from the patient administered with iodine-131” in “Materials for Calculating Exit Criteria” in the Administrative Contact (June 30, 1998), Safety Measures Division, Pharmaceutical Safety Bureau, Ministry of Health, and Welfare Estimate [19].

First, based on a report that examined air pollution caused by exhaled breath of patients who received ^131^I [24], we decided to apply the maximum volatilization rate of iodine 1.4 × 10^–5^ per hour to astatine. In addition, assuming that the volume of the room where the patient is located is 30m^3^, the ventilation rate is 1 h on average, and the caregiver's daily breathing volume is 20m^3^. And it is assumed the caregiver is always in the same room as the patient [19]. According to these assumptions, the caregiver's ingested radioactivity per 1 MBq of dose is calculated as 1 [MBq] × 1.4 × 10^–5^ [h^−1^] × (1/30) [m^−3^] × 1 [h] × 20 [m^3^/d] × 1/24 [d/h] × 1.443 × 7.214 [h]) = 4.05 × 10^–6^ [MBq].

The effective dose of internal exposure (exposure coefficient 0.5 is applied [19]) associated with inhalation per 1 MBq is 4.05 × 10^–6^ [MBq] × 10^6^ [Bq/MBq] × 2.7 × 10^–5^ [mSv/Bq] × 0.5 = 5.47 × 10^–5^ [mSv] = 0.0547 [μSv], and when 540 MBq is administered to the patient per case, the internal exposure due to inhalation by the caregiver will be 0.0547 × 540 = 29.5 [μSv].

Here, 2.7 × 10^–5^ [mSv/Bq] is the effective dose coefficient of ^211^At by inhalation [23].

Including the internal exposure from oral ingestion by the public, the total internal exposure of the caregiver is 29.5 + 0.05 = 29.55 [μSv]. This figure, 29.55 [µSv] is well below the dose constraint value of 5 mSv (5,000 µSv) per caregiver.

## Comprehensive evaluation of the external and internal exposure doses

The results of a combined evaluation of the external exposure dose and internal exposure dose that the caregiver or the public is exposed to for this therapy are shown below.

Caregiver: 72.4 [μSv] + 29.55 [μSv] = 0.102 [mSv].

General public: 36.2 [μSv] + 0.05 [μSv] = 0.036 [mSv].

Caregiver exposure doses are estimated to be 0.102 [mSv] and public exposure doses are estimated to be 0.036 [mSv], respectively. Both values meet the recommended dose criteria for each category.

This calculation is based on assuming exposure to a maximum dose of 540 MBq (up to 4 times a year), but the annual dose may increase depending on future clinical trial protocols. In that case, it is necessary to reevaluate with the maximum dose. However, since the estimated exposure doses of both the public and the caregiver are far below the dose limit, it is considered that there may be no significant effect.

## Release of patients administered [^211^At] MABG

Even if a patient leaves the medical radioisotope use room or radiotherapy room immediately after administration of [^211^At] MABG, the recommendations of the International Commission on Radiological Protection (ICRP) and the safety standards of the International Atomic Energy Agency (IAEA) are supposed to be satisfied. In addition, it is considered that the concept of the exit criteria in the "Guidelines for Exit of Patients Receiving Radiopharmaceutical Drugs" (Notice of Pharmaceutical Safety No. 70) is also satisfied.

For this reason, patients receiving [^211^At] MABG do not need to be admitted to a radiotherapy room as stipulated in Article 30-15 of the Medical Care Act Enforcement Regulations.

## Precautions for patients and their families

After administration of [^211^At] MABG, trace amounts of radioactivity are present in body fluids (mainly blood), urine and feces. Since most of this drug that was unabsorbed by the tumor is excreted from the renal and urinary tract system, the precautions illustrated below should be documented to patients and their families (caregivers). It is necessary to explain and gain understanding before administration.

(1) Precautions to be taken 2 days post-administration for patients and their families

## [Caution in daily life]

① When the patient bleeds, wipe off the blood with toilet paper and flush it down the toilet.

② Wear disposable rubber gloves before handling the patient's urine or feces, or clothing contaminated with [^211^At] MABG.

③ If the patient's blood or other body fluids come into contact with the hands or skin, wash the touched area with soap immediately.

④ Refrain from sexual intercourse.

⑤ Keep all persons living with the patient as far away as possible. It is desirable to keep at least 1 or 2 m away or more when staying for a long time. Minimize contact with children and pregnant women.

⑥ Avoid sleeping in the same bed as other people. Sleep at least 2 m away and, if possible, in a separate room.

⑦ Patients should be the last to bathe each day. In addition, after bathing scrub the bathtub thoroughly with detergent.

⑧ Refrain from going out to public places (for example, public transportation, supermarkets, shopping centers, movie theaters, restaurants, sports arenas, etc.) as much as possible.

## [Notes on handling laundry]

Clothes worn by the patient should be separated from the clothes of others, and simultaneous washing should be avoided. In addition, sheets and underwear with blood or urine should be pre-washed thoroughly.

## [Caution when urinating / defecating / vomiting]

Male patients should urinate in a sitting position.

If feces or urine spills on the toilet bowl or floor, wipe it clean with toilet paper and flush it down the toilet. (Especially if they are the patient`s.)

Flush the toilet bowl twice after use. (Especially the patient.)

Wash your hands thoroughly with soap after urinating and defecation. (Especially the patient.)

⑤ Be sure to wash your hands and skin with soap and thoroughly wash the hands and skin when you come in contact with body fluids such as the patient`s blood, excrement, or vomit.

(2) Precautions for 1-week post-administration

Female patients should avoid breast-feeding for a week. When using official facilities (border-crossings, airports, etc.) where radiation detection is performed overseas to prevent terrorism, carry a medical certificate of this therapy.

(3) Precautions for 3 months post-administration

Patients who received [^211^At] MABG should use contraception for 6 months after administration, regardless of gender.

(4) Radiation safety management for patients using diapers and urinary catheters

For patients using diapers and urinary catheters, the following precautions should be taken early after administration (about 2 days):

When handling diapers, urinary catheters, and urine storage bags, wear disposable gloves for biohazard prevention.

## [Precautions when using diapers, urinary catheters, etc. (in-home/in-hospital)]

① For patients with urinary incontinence and using diapers, it is also recommended to use vinyl sheets.

② When using a urinary catheter, discard the urine in the urine bag into the toilet, flush the water twice, and wash your hands thoroughly after treatment.

③ For inpatients, replace the urinary catheter and urine storage bag before discharge.

④ Put the replaced diapers in a plastic bag, etc., and store them in a sealed state until 3 days after administration of this drug.

## [Precautions when disposing of diapers, urinary catheters, etc.]

(1) Treated patients’ diapers used at home should be treated as general waste. They should be placed in a plastic bag and sealed so that the contents do not leak. However, if necessary, dispose of them in a manner corresponding to the disposal method of the local government.

(2) When disposing of infectious waste such as diapers in the hospital, refer to "Handling Diapers of Radiopharmaceutical-administered Patients (Guidelines for Medical Professionals Practicing Nuclear Medicine) (1st edition, March 2001)” and "Revised 2nd edition, March 2004″ [25].

## Regulatory laws governing clinical trials on [^211^At] MABG

The following are regulatory laws and regulations regarding the prevention of radiation hazards when conducting clinical trials of [^211^At] MABG.

(1) Act Regulating Radioactive Isotopes: Nuclear Regulation Authority [26].

(2) Medical Care Act [27] (Implementing regulations [28]): Ministry of Health, Labour and Welfare.

(3) Act on Securing the Quality, Efficacy, and Safety of Products Including Pharmaceuticals and Medical Devices: Ministry of Health, Labour and Welfare of Japan.

(4) Medical Practitioners’ Act: Ministry of Health, Labour and Welfare of Japan.

(5) Pharmacists Act: Ministry of Health, Labour and Welfare of Japan.

(6) Act for Medical Radiology Technicians: Ministry of Health, Labour and Welfare of Japan.

(7) Industrial Safety and Health Act (Regulation on Prevention of Ionizing Radiation Hazards [29] and Working Environment Measurement Act): Ministry of Health, Labour and Welfare of Japan.

(8) National Civil Service Act (Rules of the National Personnel Authority 10–5 [30]): National Personnel Authority.

## Standards for medical radioisotope rooms

Healthcare facilities that perform this therapy using [^211^At] MABG must have medical radioisotope rooms, storage, and disposal facilities in compliance with the standards for radiation hazard prevention stipulated in Article 30-8, Article 30-9, and Article 30-11 of the Medical Care Act Implementing Regulations.

## Standards for concentration limits in medical radioisotope rooms

For healthcare facilities that carry out nuclear medicine medical care, the structural equipment must comply with the standards such as the concentration limit shown in Table 6.

**Table 6:** Standards for dose and concentration limits for medical radioisotope rooms based on Japanese medical care act

Rooms	Medical radioisotope rooms
	Storage facilities
	Disposal facilities
	Radiotherapy rooms
Dose and concentration limits in controlled areas	Effective dose of external radiation: 1.3 mSv per 3 Months Concentration of RI in the air: The average concentration in 3 months is 1/10 of the concentration limit of the RI in the air Surface density of substances contaminated by RI: 1/10 of the surface density limit (alpha ray-emitting RI: 0.4 Bq/cm2, non-alpha ray-emitting RI: 4 Bq/cm2)
Dose and concentration limits in regular thoroughfares in RI facilities	Effective dose outside the wall: 1 mSv per week RI concentration in air: the average weekly concentration is the RI concentration limit in air Surface density of substances contaminated by RI: Area density limit (alpha ray-emitting RI: 4 Bq/cm2, non-alpha ray-emitting RI: 40 Bq/cm2)
Dose standards at the boundaries of hospitals (including residential areas inside the hospital)	Effective dose: 250 μSv per 3 months
Exposure dose of in-patients	Effective dose should not exceed 1.3 mSv per 3 months

## Restrictions on places of use

Medical radioisotopes must be handled in a medical radioisotope room. However, this limitation does not cover temporary use in the operating room after taking appropriate protective and anti-contamination measures, the radiotherapy room for patients who are difficult to transfer, or temporary use in intensive care units or heart disease strengthening treatment rooms. "Appropriate protective measures and pollution prevention measures" in the relevant regulations are described in the 4th management of 0315 No. 4 notification issued by the medical administration: Matters concerning obligations 1. (11).

## Safety management associated with the use of [^211^At] MABG at radiation facilities

(1) Management using record books, etc. (Medical Law Enforcement Regulations Article 30-23).

When using this drug, it is necessary to ensure radiation safety management, such as using it in an appropriate manner to ensure radiation safety and clarifying the location of radioactive substances by storing it in a designated place. For that purpose, it is stipulated that the following items should be kept and always managed with a usage record book, etc. [31].

(2) Records regarding acceptance, use, storage, and disposal of [^211^At] MABG (radiopharmaceutical use record book).

(The following items are required in the usage record book, based on Medical Law Enforcement Regulations, Article 30-23, Paragraph 2, Notification of Ministry of Health and Welfare, Medical Affairs Bureau, No. 51, 1974, Notification of Medical Administration No. 0315, No. 4.)

(a) product standard, (b) arrival date, (c) date of use, (d) amount used, (e) remaining amount, (f) user, (g) name of patient used, (h) storage and disposal date, (i) radioactivity at storage and disposal.

In addition, create a storage record book for stored medicines and regularly check that the storage quantity of the facility does not exceed the maximum planned storage quantity reported for each nuclide.

(3) Measurement and recording of places where radiation damage may occur (based on Medical Law Enforcement Regulations Article 30–22, Ionization Regulations Article 54).

The radioisotope use room, etc. (outside the wall of the usage room, usage room, storage room, disposal facility (storage and disposal room and drainage facility)), controlled area boundary, living area, radiation therapy room and site boundary. The amount of radiation and radioisotopes is measured once before the start of medical treatment and once every period that does not exceed 1 month after the start of medical treatment (for the designated place, the period does not exceed 6 months). Measure the status of elemental contamination and keep a record of the results for 5 years. The amount of radiation should be measured at 1 cm dose equivalent (rate) (70 μm dose equivalent (rate) in places where the 70 μm dose equivalent (rate) may exceed 10 times the 1 cm dose equivalent (rate)). The amount of radiation and the status of contamination by radioactive isotopes should be measured with a radiation measuring instrument. In principle, the 1 cm dose equivalent (rate) is measured with a radiation measuring instrument that can appropriately measure the amount of radiation emitted from the radioisotope used. However, if it is extremely difficult to measure using a radiation measuring instrument, these values can be calculated. "When it is extremely difficult to measure using a radiation measuring instrument" means "limited to cases where it is difficult to measure physically. Only in this case, calculation is permitted. As shown in the notification of 0315 No. 4 issued by Medical Affairs Bureau, it is not acceptable to apply this provision easily.

(4) Records of measurement and calculation of radiation dose for radiation medical workers, etc. (based on Medical Law Enforcement Regulations Article 30–18, Ionization Regulations Article 8)

The effective dose and equivalent dose for radiation medical workers, etc. are measured for external and internal exposure doses, and are calculated based on the results as determined by the Minister of Health, Labor and Welfare (Ministry of Health, Labor and Welfare Notification No. 398, 20).

(5) Ionizing Radiation Health Examination Individual Card (based on Ionization Regulations Article 57)

Record the results of the "ionizing radiation health examination" for workers (radiation medical workers) who are always engaged in radiation medical services in the "individual ionizing radiation health examination card".

(6) Record of release of patients who received this drug (based on Notice of Pharmaceutical Safety No. 70).

If you are allowed to leave or return home, record the following items and keep them for 2 years after release.

 Dosage, date and time of exit, dose rate measured at the time of release.

 Contents of caution and guidance for mothers with breast-feeding infants.

## Radiation measurement

Measurement of dose (radioactivity)

The measurement of the radioactivity of ^211^At regarding the dose is performed by a dose calibrator or curie meter as well as a radioactivity diagnostic agent such as ^99m^Tc or ^123^I or a radiotherapy agent such as ^90^Y, ^131^I and ^223^Ra. It is measured using a well-shaped ionization chamber. The measurement method is the same as that of conventional ones such as radioactive diagnostic agents, and ^211^At enclosed in a specified container (vial bottle) is installed at the measurement position of a well-type ionization chamber using a jig for measurement. Since ^211^At is a nuclide that has not yet been used, the well-shaped ionization chamber used may not be calibrated by ^211^At (it does not have the calibration constant of ^211^At). When measuring for the first time, it is necessary to calibrate the measuring instrument with ^211^At in advance or contact the manufacturer of the measuring instrument to set the calibration constant.

8.2 Dosimetry of the place of use, etc.

When using radioisotopes for medical treatment, the air dose in the place where people in the controlled area always enter, the boundary of the controlled area, the boundary of the site, the living area, etc., the radiation dose at the time of leaving the patient, the radiation medical staff, and the individual exposure dose of the worker must be measured regularly or as needed (see 7.1.2). ^211^At radiation control dosimetry is performed on gamma rays. The field air dose is measured with a 1 cm dose equivalent H * (10) as the ambient dose, and the exposure dose is measured with a measuring instrument calibrated with a 1 cm dose equivalent Hp (10) as an individual dose equivalent.

As a measuring instrument for measuring the air dose, a survey meter using a scintillation detector such as an ionization chamber or a NaI (Tl) scintillation detector as a detection unit is used. Ionization chambers are suitable for measurements in places with relatively high dose rates, such as places of use, and highly sensitive NaI (Tl) scintillation survey meters are effective in low-dose areas such as controlled area boundaries and site boundaries. In addition, to evaluate the cumulative dose for a certain period such as one week or three months, the cumulative dose during the period may be calculated appropriately based on the instantaneous dose rate measured by the survey meter shown above (generally expressed in μSv / h, but several to several tens of seconds). However, a measuring instrument capable of measuring the cumulative dose may be used.

There are two types of personal dosimeters, one that directly displays the exposure dose and the other that calculates the exposure dose with a reading device after wearing it for a certain period (called a passive type). The passive type is generally a personal dosimetry service. Ask the institution to read the exposure dose. Since those that directly display the exposure dose are measured by putting them in a pocket or the like, they are also called direct-reading pocket dosimeters, and recently, those using semiconductors such as Si are often used. Film badges were the mainstream for passive dosimeters, but recently fluorescent glass dosimeters and photo stimulated luminescence dosimeters have been used.

## Education and training

It is necessary to acquire knowledge about ensuring medical safety related to this treatment and the safe handling of radiation. Education and training based on this manual at each medical institution should be conducted on the following:

① Laws, notification items and exit criteria regarding radiation hazard prevention.

② Chemical and physical properties of this drug and radiation protection.

③ Exposure prevention for medical staff and instructions for patients and their families.

④ Radiation measurement and safety management of radioactive waste

Physicians who have acquired specialized knowledge through education and training conducted in the hospital can play the role of the practitioner of the therapy. In that case, it is ideal for the physician to be appointed as the manager or investigator at the hospital with which they are affiliated.

In addition, keep a record of the education and training conducted in the hospital. Implementation records shall be retained for at least 2 years.

## Radiological protection measures related to the handling of [.^211^At] MABG

(1) Preparation of protective equipment.

(a) Protective glasses (required): prepare in anticipation of the possibility that the injection will directly contaminate the eyeball in the process of handling this drug.

(b) Wear protective gloves (required): to prevent direct contamination of fingers, etc. when handling this product.

(c) Water-absorbent polyethylene filter paper: a polyethylene filter paper that absorbs water containing radioactive substances and prevents the spread of pollution. Cover the inside of the potentially contaminated safety cabinet, the work surface around it, and lead blocks with polyethylene filter paper.

(d) Tweezers: when a silicon tube or the like is attached to the tip of the tweezers, it acts as a non-slip surface? and makes it easy to grab the vial or the like with the tweezers.

(e) Appropriate size vat: if a water-absorbent polyethylene filter paper is placed on an appropriate size stainless-steel vat and dispensing work from the drug vial to the injection syringe is performed on this vat, even if a liquid containing radioactivity spills during operation, radioactive contamination can be retained in the vat, helping to prevent the spread of contamination.

(2) Basics regarding the handling of radioactive substances.

Attention should be paid to the handling of radiopharmaceuticals, which are unsealed RIs, as well as external exposures, as well as internal exposures resulting from their uptake into the body. Also, unlike sealed RIs, radiopharmaceuticals are often handled at close range. In addition, it should be considered that post-administration patients are also sources of radiation exposure. Therefore, when handling this drug, try to reduce the exposure by shortening the working time, keeping a distance from the radiation source, and providing a shield (three principles of external exposure protection).

(a) Implementation of cold run (practice of operation to handle [^211^At] MABG).

Regarding the actual procedure using a vial containing this drug, a dispenser, etc., the act of performing the same procedure as when using RI without using radioactive substances (RI) is called cold run.

The work procedure can be checked and understood by repeating this work, practicing, and becoming skilled.

The preparation of necessary equipment and protective parts can be checked.

It helps to reduce mistakes by speeding up the work of operating with actual radioactive substances. That is, it is possible to speed up the work of handling the radiation source (shorten the time) and reduce operation mistakes such as mistakes in the procedure.

(b) Precautions in the controlled area

Precautions for entering and exiting controlled areas and laboratories should be posted near the entrance and exit as a matter of compliance with the Medical Care Act. Therefore, radiation medical (medical) workers involved in radiation work need to be thoroughly aware of these precautions. The main precautions are shown below:

## 1 Keep an entry record.

② Radiation clinic workers should change into slippers, athletic shoes, safety shoes, etc. exclusively for the controlled area.

③ Radiation medical staff should change into work clothes exclusively for the controlled area.

④ Wear a personal exposure dosimeter such as a pocket dosimeter on the chest for men and on the abdomen for women.

⑤ Make sure that the ventilation system of the exhaust equipment is operating.

⑥ Be sure to wear protective glasses and gloves when handling radiopharmaceuticals.

⑦ After use, dispose of radiopharmaceuticals and radioactive substances, move them to the storage and disposal room immediately after the work is completed.

⑧ After use, the room will be inspected for radioactive contamination, and if it is found to be contaminated, it will be immediately decontaminated.

⑨ Wash your hands with detergent and running water.

⑩ Inspect hands, feet, cuffs, clothing surface, footwear, etc. for contamination.

⑪ If there is no contamination, change your clothes. If contamination is found, decontaminate according to the instructions of the radiation administrator.

⑫ Keep a record of leaving the room.

⑬ Read and record the value of the personal exposure dosimeter.

(3) Handling of [^211^At] MABG.

Dispensing work of [^211^At] MABG: generally, when the dose of [^211^At] MABG is reduced, the dispensing work is required, and this dispensing work should be performed in the safety cabinet. Make sure the safety cabinet is working properly. In addition, the floor near the safety cabinet is covered with polyethylene filter paper so that it can be easily decontaminated, and if necessary, the work surface inside the cabinet, the back of the front and the sides are also covered with polyethylene filter paper. In addition, when handling radiopharmaceuticals, a shield such as a lead plate or a block is used for medical personnel to reduce their exposure to radiation.

Administration of [^211^At] MABG: this drug should be administered slowly intravenously. When administering this drug, take measures to control exposure and contamination of radiation medical personnel.

Procedures for handling [^211^At] MABG and disposing of waste after administration: when handling this drug, use protective glasses. Also, wear protective equipment such as lab coats and gloves. Work with this agent, etc. in a stainless-steel vat covered with water-absorbent polyethylene filter paper, etc. The same applies to the work of pollutant treatment. If the surface of the skin such as the face or the eyeballs is contaminated with this product, immediately wash thoroughly with detergent and running water.

Radiation medical practitioners should not leave or walk around while performing radiation work such as preparing medicines. Immediately after the work is completed, the waste is separated and stored for disposal.

Contamination inspection and decontamination of rooms, etc. (walls, floors, etc.) using this agent: check the presence or absence of contamination by this agent in the safety cabinet, floor, etc. along the flow line using this agent. It is needed to measure using a radiation measuring instrument.

Since ^211^At emits α-rays and X-rays, it is important to use a radiation measuring instrument that is effective in measuring ^211^At for detecting surface contamination. Simultaneous preparation and dispensing of other nuclides of pharmaceuticals in the room may lead to misadministration, etc., and should be avoided as much as possible from the viewpoint of ensuring medical safety.

Generally, it is desirable to measure alpha rays with a ZnS (Ag) scintillation survey meter as the measuring instrument when measuring the spots contaminated by ^211^At on the workbench or floor but counting with sensitivity to X-rays around 80 keV. A NaI (Tl) scintillation survey meter capable of rate counting and a GM survey meter sensitive to 12.4 keV Po-L X-rays (see Table 1) can also be used simultaneously.

If radioactive contamination is found on the workbench or floor, it is necessary to promptly decontaminate it. If contamination is found relatively early, the general procedure is to absorb it with a paper towel or the like and gradually decontaminate it with water, a neutral detergent, a chelating reagent such as citric acid, or the like. When decontaminating, pay attention to cracks and pinholes in the gloves used to prevent secondary contamination of the body. If complete decontamination is not possible, mark the extent of contamination, measured values ​​and the date of contamination with magic ink to clarify the contaminated area. In addition, preventing the spread of pollution by preventing the spread of pollution by measures such as prohibition of entry is also an appropriate method of radiation exposure prevention and pollution prevention measures.

## Exposure of medical personnel (external and internal exposure)

Managers of healthcare facilities are responsible for preventing exposure of medical personnel in accordance with Articles 30–18 and 30–27 of the Medical Care Act Implementing Regulations, Items 1 to 2 related to No. 5: limitations in the HPB 0315, No. 4 Notice and 1 to 5 in No. 6: Calculation of dose.

The maximum single dose for this treatment is 540 MBq, and the external exposure dose of healthcare workers is calculated as shown in Table 7 based on the relationship between working time and distance from the radiation source. For dose evaluation, 0.00644 [μSv m^2^ MBq^−1^ h^−1^] is used as the effective dose rate constant, which is calculated as the sum of the effective dose rate constant of 211At described in the 12th edition of the Isotope Handbook and the constant of the progeny nuclide.

**Table 7:** The external radiation dose to health care workers

Stage of work	Effective dose (per case)			Skin dose ^a^(per case)			Dose limit	
	Working time (min)	Distance (cm)	Exposure dose (mSv)	Working time (min)	Distance (cm)	Exposure dose (mSv)	Effective dose limit (whole body)	Equivalent dose limit (skin)
Preparation	10	50	0.0023	10	10	0.058	Radiological professionals: 50 mSv/year, 100 mSv/5 years Women who can be pregnant: 5 mSv/3 months	500 mSv/year
Administration	5	50	0.0012	5	5	0.116		

^a^Reference value using effective dose rate constant. The skin equivalent doses should be measured by 70 µm dose equivalents

The effective dose (mSv/week), *E* due to internal exposure of workers per week is calculated by the following formula based on "December 26, 2000, Ministry of Health and Welfare Notification No. 398, [23])".

*E* = e × *I.*

Here, *I* is the quantity (Bq) of medical radioisotopes inhaled per week.

*I* = 1.2 × 10^6^ × *C*  ×  *t.*

1.2 × 10^6^: Intake of air inhaled by an adult per hour (cm^3^/h)

*C*: Average radioactivity concentration in the air per week (Bq/cm^3^).

*t*: Working hours/week.

*C* = *A* × scattering rate × number of days used per week / (*V* × 10^6^ × 8 (h) × 1 week exhaust equipment Working days).

*A*: Maximum planned daily quantity (Bq).

*V*: Indoor displacement (m^3^/h).

It shall be operated for 8 h / day with a displacement of V (m^3^/h).

In the case of this drug, *A*: 540 MBq, scattering rate: 0.001, daily indoor displacement: 560 (m^3^/h) × 8 (h), number of days of use per week: 1 day (number of days of use of this drug), weekly exhaust facility operating days:

5 days, working time: 10 min (0.167 h), e (effective dose coefficient when ^211^At is inhaled): 2.7 × 10^–5^ (mSv/Bq). The effective dose *E* (mSv) due to internal exposure per week is as follows:

*C* = 540 × 10^6^ × 0.001 × 1 / (560 × 10^6^ × 8 × 5) = 2.41 × 10^–5^ (Bq/cm^3^).

*I* = 1.2 × 10^6^ × *C* × 0.167 × 1 = 4.83 (Bq).

*E* = *e* × *I* = 2.7 × 10^–5^ × 4.83 = 1.3 × 10^–4^ (mSv).

## Precautions for medical personnel

Medical professionals involved in nuclear medicine treatment with this drug should fully understand this manual and the pharmacokinetics of this drug, and then explain the above-mentioned principles regarding radiation protection to patients and their families in an easy-to-understand manner. In addition, doctors with specialized knowledge regarding this treatment should provide appropriate education and training to medical professionals and strive to enhance the cooperation system at the medical institutions concerned. If urgent medical treatment is required, appropriate medical treatment may be prioritized over the above-mentioned compliance items regarding radiation protection to secure the lives of patients and the like.

Especially for those who are engaged in patient care, pay attention to the following points for 1 week after administration:

(1) Wear disposable rubber gloves when there is a possibility of contact with the patient's urine, feces, or blood, or when handling clothing contaminated with these.

(2) If there is contact with the patient's excrement or blood, be sure to immediately wash the contaminated parts such as hands and skin with soap and thoroughly wash with water.

(3) Wash clothes contaminated with patient excrement or blood separately from other people's clothes.

## Disposal of medical radioactive contaminants (contaminated with ^211^At)

Substances contaminated with [^211^At] MABG fall under "medical radioactive contaminants" as stipulated in Article 30-11 of the Medical Law Enforcement Regulations. Medical radioactive contaminants should be stored and disposed of at the "disposal facility (storage and disposal facility)" in hospitals, etc. based on the provisions of Article 30-11 of the same Act. In addition, inquire of the person designated to be entrusted with the disposal of the medical radioisotope or the substance contaminated by the radioisotope as stipulated in Article 30-14-2, Clause 1 of the Act.

When handling human excrement in diapers and urine bags and items contaminated with blood, please refer to Handling Diapers of Patients administered Radiopharmaceuticals (Guidelines for Medical Professionals Practicing Nuclear Medicine) and “Handling manual for diapers of patients administered radiopharmaceuticals” [25].
